# Imaging evaluation of peripheral nerves

**DOI:** 10.1590/0100-3984.2023.56.1e2

**Published:** 2023

**Authors:** Clarissa Canella

**Affiliations:** 1 Adjunct Professor of Radiology, Universidade Federal Fluminense (UFF), Niterói, RJ, Brazil; Musculoskeletal Radiologist at the Clínica de Diagnóstico por Imagem (CDPI) and at the Clínica Alta Excelência Diagnóstica, Rio de Janeiro, RJ, Brazil. Email: clacanella@yahoo.com.br.

Ultrasound is an important imaging method for the evaluation of peripheral nerves. The use of a combination of clinical and electroneuromyography data often makes it possible to identify a specific neurological disorder. In the upper extremities, some of the most common neurological disorders are carpal tunnel syndrome, cubital tunnel syndrome, and cervical radiculopathy. Although in many cases magnetic resonance imaging (MRI) continues to be the gold standard for nerve imaging, especially for the identification of deep nerves, ultrasound has become an essential imaging technique because of its ability to offer dynamic information, its more widespread availability, and its lower cost (in comparison with MRI)^(1)^.

I totally agree with Voltan et al.^(2)^, as published in the previous issue of Radiologia Brasileira, that high-resolution ultrasound provides information complementary to that obtained by electroneuromyography, especially on the morphology of nerve fascicles and the vascularization of peripheral nerves. Those authors also stated that, as well as being noninvasive and rapidly executed, high-resolution ultrasound can be used in order to assess neural dimensions, locate compression zones within fibrous tunnels, identify tumors, and diagnose traumatic pathologies^(2)^. Given the lack of data on this topic in the literature, it is important that the authors demonstrated that the peripheral nerve cross-sectional areas were lower in a sample of the Brazilian population than in most of the other samples described in the literature. On the basis of their findings, Voltan et al.^(2)^ proposed cross-sectional area reference values for the Brazilian population: for the median nerve (in the carpal tunnel); for the ulnar nerve (at the pre-cubital tunnel site); and for the common fibular nerve (near the fibular head). Those reference values will certainly help radiologists in daily practice.

Although ultrasound and MRI are currently the gold standards for peripheral nerve analyses, I would like to highlight the importance that magnetic resonance neurography (MRN) has taken on in recent years. It is a technique that improves selective multiplanar visualization of the peripheral nerves and of nerve pathologies by combining two-dimensional, three-dimensional (3D), and diffusion imaging pulse sequences. Images acquired with MRN provide a highly accurate depiction of the relationships between lesions and the nerve architecture, and the technique makes it possible to visualize neuromuscular alterations directly, thus facilitating an objective evaluation of the consequences of neuromuscular pathology^(3)^. In addition, high-resolution isotropic volumetric 3D MRN enables multiplanar depiction of peripheral nerves, as well as allowing anatomical and functional characterization of various neural diseases^(4)^. In summary, imaging techniques ranging from ultrasound to high-resolution isotropic 3D volumetric MRN are essential to evaluate conditions and diseases affecting the peripheral nerves, including entrapments, trauma, inflammatory or infectious neuropathies, and neoplasms.
